# Total Joint Replacement in the Past Does Not Relate to a Deteriorated Functional Level and Health Status in the Oldest Old

**DOI:** 10.1155/2012/968389

**Published:** 2012-05-08

**Authors:** Wiebe Chr. Verra, Anton J. M. de Craen, Coen C. M. M. Jaspars, Jacobijn Gussekloo, Gerard Jan Blauw, Rudi G. J. Westendorp, Andrea B. Maier, Rob G. H. H. Nelissen

**Affiliations:** ^1^Department of Orthopaedic Surgery, Leiden University Medical Center, P.O. Box 9600, 2300 RC Leiden, The Netherlands; ^2^Department of Gerontology and Geriatrics, Leiden University Medical Center, P.O. Box 9600, 2300 RC Leiden, The Netherlands; ^3^Department of Public Health and Primary Care, Leiden University Medical Center, P.O. Box 9600, 2300 RC Leiden, The Netherlands; ^4^Netherlands Consortium for Healthy Ageing, Leiden, The Netherlands

## Abstract

Total hip or knee replacement is effective in improving joint function, quality of life, and pain reduction. The oldest old population with joint replacements (TJR) is underrepresented in current literature. We compared health-related and functional characteristics of oldest olds with and without TJR. Participants (aged 85 years) were divided into a group with and without TJR. Comorbidity, physical and joint functioning, daily living activities, quality of life, and mortality were recorded. Thirty-eight of 599 participants (6.3%) received a TJR in the past. Participants with a TJR had slightly less comorbidities, walked slower (*P* = 0.006), and complained more about hip-pain (*P* = 0.007). Mortality of those with a TJR was lower during the first 8-year followup (*P* = 0.04). All other characteristics were comparable between groups. We conclude that subjects with a TJR performed equally well, besides showing a lower gait speed and a higher frequency of hip-pain. Except for the lower gaitspeed, having a TJR is not associated with poorer health.

## 1. Introduction

The population of oldest olds (i.e., 85 years and older) is the fastest growing segment of the elderly population in the western society [1]. The health status decreases with increasing chronological age [2]. One of the major age-related diseases is osteoarthritis (OA), which is more common in females [3–5]. In subjects between 60 and 70 years of age, prevalences of symptomatic knee OA are reported of approximately 10 percent in males and 20 percent in females [4]. Prevalence of knee OA is comparable in subjects aged 80 years and older [4, 5]. Symptomatic OA of the hip is present in approximately five percent of the 60 to 70 years old females and up to 18 percent in females of 80 years and older. In males, prevalences are slightly lower [3, 4]. Due to the demographic changes, the number of total hip replacement (THR) and total knee replacement (TKR) procedures steadily increases [6]. Increasing age is associated with a higher complication and mortality rate after total joint replacement [6]. However, the results of total joint replacement in elderly patients have been proven effective in terms of pain reduction, functional improvement, and cost-effectiveness and show similar results compared to younger patients receiving total joint replacement [7, 8].

OA of the hip or the knee impairs physical activity [4]. Restriction of physical activity is associated with numerous detrimental effects on general health status, physical function, and quality of life [4, 9]. Maintaining physical activity at older age is essential in order to maintain optimal health status. Treating OA, ultimately with a total joint replacement, influences function (i.e., flexion, extension, rotations) and quality of life positively [8]. However, in terms of improving physical activity level, the influence of a total joint replacement is less clear [10]. The long-term effects of receiving a total joint replacement have been underrepresented in the oldest old population.

In the present study, we compared a group of oldest old subjects with and without a total hip or knee replacement in their history. Since surgery is performed preferably on healthy subjects and based on the aforementioned positive effects of total joint replacement, we hypothesized that the group with total joint replacement would show better results on physical functioning, daily living activities, joint complaints, and quality of life.

## 2. Methods

### 2.1. Participants

All data originate from the Leiden 85-plus Study, a community-based prospective follow-up study of inhabitants of the city of Leiden, the Netherlands. All participants were included at the age of 85 years. There were no exclusion criteria. Follow-up visits were performed annually. Enrolment of the study took place between 1997 and 1999 [11]. A total of 599 persons participated in the study, 87% of all eligible inhabitants. The Medical Ethical Committee of the Leiden University Medical Center approved the study. Informed consent was obtained from all participants.

### 2.2. Total Joint Replacement

In order to determine whether participants had received an elective THR or TKR, medical history concerning total joint replacement was obtained from the hospital charts and from information provided by general practitioners and nursing home physicians.

### 2.3. Participant Characteristics

Physical functioning was assessed at the participant's home, by the following items: if a participant was able to stand up and walk, gait speed, a five times stand-up test, hand grip strength, and a physical activity score. The ability to stand up and to walk was recorded dichotomously. Gait speed was determined by using the six meter walking test [12]. Use of a walking aid was allowed. Gait speed was calculated using distance in meters and time in seconds (m/s). In the five times stand-up test participants were asked to stand up five times in a row, from sitting. Time was recorded in seconds. Hand grip strength, as a proxy of muscle strength, was measured with a Jamar hand dynamometer (Sammons Preston Inc. Bolingbrook, IL). Participants were asked to stand up and hold the dynamometer in the dominant hand. After one trial, participants were asked to squeeze three times. The maximum measurement was recorded in kilograms (kg).

To calculate the physical activity score (PAS), four items from the Time Spending Pattern questionnaire were selected to constitute physical exercise above routine daily physical activity [13]: (a) walking for fun, (b) cycling for fun, (c) exercise alone or in groups or other physical activity, and (d) working in the garden. Each item was scored from 0 (no activity) to 3 (daily activity), and their sum score made up the Physical Activity Score (PAS).

 Activities of daily living were measured using the Groningen Activity Restriction Scale (GARS) [14]. The GARS assesses competence in abilities in nine personal basic activities of daily living (ADL) and nine instrumental activities of daily living (IADL). A summed score was calculated for basic IADL ranging from 9, indicating ability to perform all activities without assistance or undue effort, to 36 indicating disability. To assess joint complaints, participants were asked whether they experienced pain and stiffness of any hip or knee joint.

Quality of life was assessed with the Cantril ladder [15]. This quality of life-score uses a ten-point scale ranging from 0 “worst possible life” to 9 “best possible life.” Furthermore participants were asked to qualify their health status; results were dichotomised between good and poor.

### 2.4. Other Characteristics of Participants

Participants' gender, demographics, socioeconomic status, marital status, and education were recorded. Body mass index (BMI) was calculated as weight in kilograms divided by height in meters squared. Chronic diseases identified from general practitioner and pharmacists' records included cardiovascular disease (CVD), including myocardial infarction, angina pectoris, and hypertension. Furthermore, diabetes mellitus, obstructive pulmonary disease, Parkinson's disease and arthritis (including rheumatoid arthritis and osteoarthritis) were recorded. Numbers of prescribed medicines were recorded from pharmacists' records. Global cognitive performance was assessed with the Mini-Mental State Examination (MMSE) [16]. Furthermore the 15-item Geriatric Depression Scale (GDS-15) was used to measure depressive symptoms [17]. This scale is developed to determine depression in the elderly and is filled in by the participants themselves. A score of six or more indicates the possible presence of depressive symptoms. Because of limited validity of the GDS-15 in people with moderate and severe cognitive impairment, it was completed only by people with MMSE scores of more than 18.

### 2.5. Statistics

For continuous data means with standard deviations and for non-Gaussian data medians with interquartile ranges were calculated. Differences between the two groups were calculated using the *t*-test when data was continuous, Mann-Whitney-*U* test for nonparametric data, and chi-square test when data was dichotomous. Linear or logistic regression was performed to adjust for gender. Nonparametric data was log transformed in order to obtain a normal distribution. Patient survival was analysed using the Kaplan-Meier method. Cox regression analysis was used to compute a hazard ratio comparing subjects with a THR or TKR with subjects without a joint replacement.

*P* values less than 0.05 were considered to be significant. All statistical analyses were performed using SPSS for Windows (SPSS Inc, Chicago), version 17.

## 3. Results

From the 599 participants, 38 (6.3%) were identified with 49 total joint replacements: 29 total hip replacements (THR) and 20 total knee replacement (TKR). The mean age of the subjects during their first primary joint replacement was 78.2 (SD 4.7) years. Characteristics of participants at 85 years are shown in Table 1. The prevalence of comorbidities was slightly lower in the group of participants with a joint replacement in the past compared to the group of participants without a total joint replacement. There were no statistically significant differences found between the two groups on any parameter except for the prevalence of arthritis. From the 38 participants, 28 had one total joint replacement. Five had 2 TKR's and three had 2 THR's, one had both a TKR and a THR, and one had a THR and 2 TKR's.

### 3.1. Physical Functioning, Daily Living Activities, Joint Complaints, and Self-Reported Health

Table 2 shows the functional characteristics of the participants with and without joint replacement at age 85 years. In both groups, most of the participants were able to walk. Participants with a total joint replacement walked significantly slower compared to participants without joint replacement (*P* = 0.006). All other tested items addressing physical functioning were similar between both groups. In terms of daily activities and self-reported health status, there were also no differences between both groups.

The number of participants with a total joint replacement complaining about hip pain was significantly higher compared to the number of participants without a joint replacement (*P* = 0.007). Within those complaining of hip pain, 11 participants had received at least one THR and four participants had at least one TKR in the past. The number of participants complaining about knee pain differed between both groups; however, this result did not reach statistical significance (*P* = 0.06). Within those complaining of knee pain, 8 participants had received at least one TKR and nine participants had at least one THR. Within those complaining about both hip and knee pain, 9 had received a TKR and 14 a THR.

From the participants with a THR (*N* = 26), 42% complained about hip pain and 35% about knee pain. From the participants with a TKR (*N* = 14), 29% complained about hip pain and 57% about knee pain.

### 3.2. Survival

**Figure 1:**
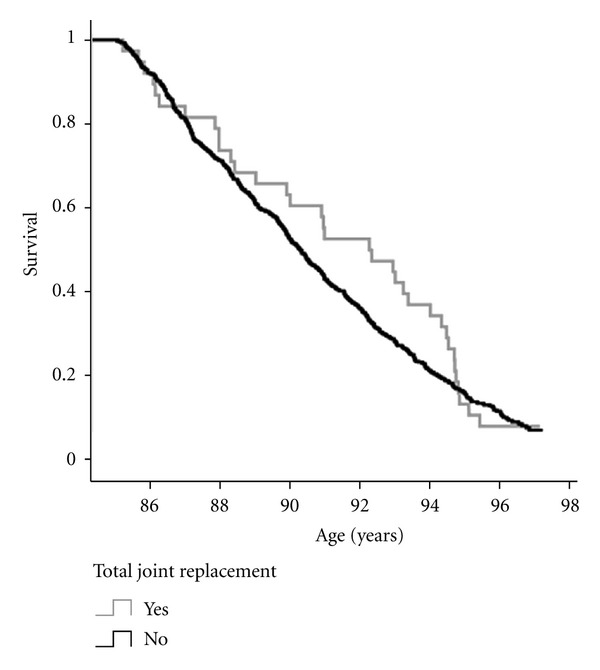
Mortality of participants with and without a total joint replacement in the past.

During a total follow-up period of 12 years (median 5.8 years, interquartile range 3.1–8.9 years), 542 (90.2%) participants died.  Figure 1 shows the Kaplan-Meier survival curve of participants with and without joint replacement. During the first 10 years, mortality was attenuated in the group of participants with a joint replacement. When applying Cox regression to calculate a hazard ratio (HR) adjusted for gender, no significant differences in survival were found after followup of 12 years dependent on the history of joint replacement (HR of 0.86, 95%-CI [0.61, 1.22], *P* = 0.41). Cox regression up to eight years of follow-up showed a survival benefit of the participants with a joint replacement (HR of 0.60 (95%-CI [0.37, 0.98], *P* = 0.04).

## 4. Discussion

Within the present study, characteristics of the oldest old with and without a total joint replacement in the past were compared. No differences in the prevalence of chronic, age-related diseases were found between the two groups except for the prevalence of arthritis. No differences in physical functioning were found except for a lower gait speed in the group with a total joint replacement. This group also complained more about joint pain. Furthermore, an attenuated mortality rate during followup was observed in this group.

Gait speed is considered to be an important predictor of functional status and adverse health events [18, 19]. It is also related to functional activities, such as crossing the street [19]. A recently published study confirmed our results of lower gait speed in subjects with a total joint replacement [19]. That study showed slower gait speed in middle aged to elderly patients who received a total hip replacement about 2.5 years before [19]. More severe joint pain is associated with lower gait speed in patients with osteoarthritis (OA) [20]. The group with total joint replacement complained more of joint pain; this could have contributed to the lower gait speed. It was not recorded whether the joint pain complaints came from the left, right, or both sides. A reason why oldest old participants with a joint replacement complained more about joint pain can be the presence of OA in the other lower extremity joints. Since total joint replacement is the end-stage treatment of OA, other joints are likely to be affected by OA as well [21, 22].

With our data, we could not perform a cost-effectiveness analysis for total joint replacement in the oldest old. Literature on cost-effectiveness in the general OA population shows that both total hip and knee replacement are (highly) cost-effective [23, 24]. A smaller study shows cost-effective health outcomes of total hip or knee replacement in subjects aged 80 years or older [25].

Reported quality of life did not differ between both groups in our cohort. This is in line with the results of several studies presented in a systematic review of the literature showing that subjects who received a THR or TKR performed similar in terms of health-related quality of life, as health-and age-matched controls [26]. Self-reported health status did also not differ between both groups in our cohort. There is evidence that self-reported health status improves after receiving a total joint placement in middle-aged subjects [27]. To our knowledge, this is the first study reporting on self-reported health status in oldest old subjects with a total joint replacement compared to age-matched controls after followup of, on average, seven years.

The group of participants with a total joint replacement showed a trend towards a healthier phenotype, especially in terms of cardiovascular and pulmonary diseases and attenuated mortality rate, but differences did not reach significance. Elective surgery such as total joint replacement is preferably performed on subjects with a low number of comorbidities [28, 29]. This could explain the difference in comorbidity and survival between both groups. Oldest olds with a poor physical condition might never have reached the age of 85 years and hence, were not included in our study. If these subjects were operated on despite their lesser health status, they probably died before inclusion in the study (i.e., before they reached the age of 85 years).

A limitation of the study is that no detailed information about the joint replacement surgery, such as surgical technique and prosthesis design, data from the hospital admission, and adverse events (i.e., complications) was available. The presence of a joint replacement was recorded in the study; however, the site of replacement was not consequently recorded. This data was not retrieved for al participants. Another limitation is the lack of information about the status of OA joints (i.e., radiological degree of OA) in lower extremities in both groups and the extent to which the TJR contributes to functional level. OA status can be graded based on the radiological appearance [30, 31]. However, high-grade radiological OA is a modest indication for total joint replacement since there is a poor correlation between radiological and clinical OA [5]. Furthermore, the most important factor in deciding to perform a total joint replacement is enough pain [32].

Furthermore, the average age of participants with a joint replacement was higher compared to the general average age for receiving a THR being 70 to 75 years old, and for TKR being around 70 years old [33, 34]. An explanation could be that, by retrospectively retrieving data on joint replacement, not all implants were identified. Another reason can be that a group of subjects with a joint replacement deceased before reaching the age of 85 years.

To our knowledge, this is the first study comparing an 85-year-old population who received a THR or TKR with their contemporaries who did not receive joint replacement surgery emphasising on physical functioning, joint complaints, and reported health status. Current literature concerning total joint replacement in the oldest old patient is mostly observational, describing patient satisfaction and complications in cohorts of elderly patients who received a THR or TKR [6, 28]. Some studies compare the outcome after surgery with a cohort of younger patients [29, 35]. Several case-control studies have been published; however, controls were matched based on gender, comorbidity, and surgery type rather than based on age-matched comparison [7, 36]. Another strength of our study is that the participants are part of a large longitudinal population-based cohort study with extensive measures for functioning and health with a followup of twelve years.

Future research should focus more on the growing oldest old population. Based on our study, we observed no differences in most clinical parameters in subjects aged 85 years with and without a joint replacement where those with a joint replacement walked slightly slower. Future studies should focus on gait parameters and physical functioning of the oldest old with and without joint replacement in order to further assess the impact of having a joint replacement at old age.

## 5. Conclusion

Oldest old participants with a joint replacement walked slower and complained more of joint pain compared to those without a joint replacement of the same age. Furthermore, the groups were comparable in terms of physical functioning, activities of daily living, and quality of life. Hence, having received a total hip or knee replacement is not associated with poorer functional level and health status except for a lower walking speed in those with a joint replacement, compared to subjects without a total joint replacement, which might be due to the direct effect of arthritis on gait parameters.

## Figures and Tables

**Table 1:** Baseline characteristics of participants aged 85 years with and without total joint replacement in the past.

	Total joint replacement	
	Yes (*N* = 38)	No (*N* = 561)
Female (number, %)	27 (71)	369 (66)
Widowed (number, %)	23 (61)	322 (57)
Education: primary school only (number, %)	22 (58)	331 (59)
Living situation		
Independent (number, %)	25 (66)	304 (54)
Sheltered (number, %)	5 (13)	155 (28)
Institutionalised (number, %)	8 (21)	102 (18)
Clinical characteristics		
Body Mass Index (mean, SD)	27.6 (4.5)	27.1 (4.5)
Mini Mental State Examination (points, median, IQR)	27 (25–28)	26 (22–28)
Geriatric Depression Scale (points, median, IQR)	1.5 (0–2)	2 (1–3)
Total number of medicines (median, IQR)	3 (2–5.5)	3 (1–5)
Comorbidity		
Stroke (number, %)	1 (3)	47 (8)
CVD* (number, %)	23 (61)	380 (68)
Diabetes mellitus (number, %)	3 (8)	82 (15)
Parkinson (number, %)	0 (0)	11 (2)
COPD (number, %)	1 (3)	64 (11)
Arthritis^$^ (number, %)	34 (92)	144 (28)

IQR: Interquartile Range, CVD: Cardiovascular Disease. COPD: Chronic Obstructive Pulmonary Disease,*CVD included myocardial infarction, angina pectoris, and hypertension. Arthritis included rheumatoid arthritis and osteoarthritis. ^$^ *P* < 0.001.

**Table 2:** Health and functional characteristics of participants with and without a total joint replacement in the past.

	Total joint replacement		*P* value	
	Yes (*N* = 38)	No (*N* = 561)	Crude	Gender adjusted
Physical functioning				
Able to walk (number, %)	34 (90)	492 (88)	0.75	0.69
Gait speed (m/s, mean, SD)^*£*^	0.42 (0.18)	0.53 (0.22)	0.003	0.006
5x stand up test (sec, median, IQR)^§^	15.9 (12.0–18.8)	13.6 (10.8–17.8)	0.31	0.31^¥^
Grip strength (kg, mean, SD)	21.4 (9.0)	22.7 (8.9)	0.41	0.69
Physical activity score (points, median, IQR)	3 (1–6)	3 (0–4)	0.12	0.11^¥^
GARS				
ADL (points, median, IQR)	10.5 (9–14)	10 (9–15)	0.68	0.74^¥^
IADL (points, median, IQR)	18.5 (13–25)	18 (12–27)	0.93	0.98^¥^
Joint complaints				
Pain hip (number, %)	15 (40)	91 (16)	0.004	0.007
Pain knee (number, %)	16 (42)	123 (22)	0.05	0.06
Stiffness hip (number, %)	8 (21)	70 (13)	0.63	0.59
Stiffness knee (number, %)	11 (29)	94 (17)	0.50	0.55
Self reported status				
Cantril ladder (point, mean, SD)	7.8 (1.5)	7.5 (1.8)	0.35	0.35
Self reported health “good” (number, %)	26 (88)	392 (70)	0.71	0.70

^¥^Adjustment for gender after log transformation of nonparametric variables. ^*£*^ *N* = 526, *N* with prosthesis = 34. ^§^ *N* = 450,  *N* with prosthesis = 28. SD: standard deviation. IQR: interquartile range. GARS: Groningen Activity Restriction Scale. (I) ADL: (Instrumental) activities of daily living.
